# Multisite transcranial direct current stimulation associated with cognitive training in episodic memory and executive functions in individuals with Alzheimer’s disease: a case report

**DOI:** 10.1186/s13256-021-02800-x

**Published:** 2021-04-22

**Authors:** Letícia Zanetti Marchi, Rayssa Gabriela Dantas Ferreira, Gabriella Nayara Siqueira de Lima, Jessyca Alves Silvestre da Silva, Daniel Marinho Cezar da Cruz, Bernardino Fernandez-Calvo, Suellen Mary Marinho dos Santos Andrade

**Affiliations:** 1 Department of Occupational Therapy, Universidade Federal da Paraíba, Campus I, Cidade Universitária, João Pessoa, PB CEP: 58051-900 Brazil; 2 Department of Occupational Therapy, Universidade Federal da Paraíba, Campus I, Cidade Universitária, João Pessoa, PB CEP: 58051-900 Brazil; 3 Department of Occupational Therapy and PostGraduate Program in Occupational Therapy, Universidade Federal de São Carlos, Rod. Washington Luís km 235-SP-310, São Carlos, SP CEP 13565-905 Brazil; 4 Postgraduate Program in Cognitive Neuroscience and Behavior, Universidade Federal da Paraíba, Campus I, Cidade Universitária, João Pessoa, PB CEP: 58051-900 Brazil

**Keywords:** Alzheimer’s disease, Multisite transcranial direct current stimulation, Episodic memory, Cognitive training, Executive functions, Case report

## Abstract

**Background:**

Dementia is among the most common chronic noncommunicable neurodegenerative diseases. In the long term, it causes disability and loss of autonomy and independence. It is estimated that there are 35.6 million people with Alzheimer’s disease worldwide. Several clinical aspects of this disease have been widely studied, but the main focus of study has been memory loss, which is one of the first symptoms. The present study proposes an innovative intervention that combines cognitive training and multisite transcranial direct current stimulation, which interferes with other clinical aspects of the subject.

**Case presentation:**

In this study, we present two subjects diagnosed with mild Alzheimer’s disease. Subject 1 is an 82-year-old Brazilian Latin American woman with a high school education who was diagnosed with Alzheimer’s disease 8 years ago and uses an Exelon patch. Subject 2 is an 88-year-old Brazilian Latin American woman with an incomplete primary education who was diagnosed with Alzheimer’s disease 1 year ago and received medical orientation to temporarily discontinue medications for Alzheimer’s disease. Both participants were subjected to intermittent cognitive training sessions and concomitant transcranial stimulation in three weekly 30-minute sessions in which a brain area was stimulated every 10 minutes for a total of 24 sessions, with a 2-month follow-up. Transcranial stimulation was applied to six different regions of the cortex: the dorsolateral prefrontal cortex bilaterally, the somatosensory association cortex bilaterally and Broca’s and Wernicke’s areas. Comparing the results of tests performed before and after the treatment period, a 1-point improvement was observed for both subjects on the Word Recall task of the Alzheimer Disease Assessment Scale, which evaluates symptoms related to the decline of episodic memory. Improvement in the executive functions domain was also observed through the results of the Stroop test, Victoria version.

**Conclusions:**

The results from the two presented cases show that multisite transcranial stimulation associated with cognitive training is an effective adjuvant method for the treatment of patients diagnosed with mild Alzheimer’s disease. Its effects can benefit patients’ daily routines by reducing cognitive deficits by keeping intact areas active and/or compensating for lost functions.

*Trial registration* NCT02772185. Registered 13 May 2016, http://www.clinicaltrials.gov/ct2/show/NCT02772185. Retrospectively registered.

## Background

At present, Brazil has more than 29 million people over the age of 60 years; additionally, according to data from the Brazilian Institute of Geography and Statistics (IBGE), is it believed that almost 2 million people have dementia, of which approximately 40–60% of cases are of the Alzheimer’s type.

The cognitive decline in Alzheimer’s disease (AD) is progressive and is accompanied by several behavioral and neuropsychiatric disorders [1]. It is a primary and atrophic disease due to diffuse cortical atrophy [2], which is characterized by intense synaptic loss and degeneration of the central nervous system, in addition to senile plaques and neurofibrillary tangles. Its onset is slow and gradual and results in the loss of cognitive skills, affecting social relationships and day-to-day activities [3].

AD is classified into three stages of evolution: mild, moderate, and severe. According to Santos and Borges [4], the mild phase is characterized by decreased execution of instrumental activities of daily living (IADLs), in which the individual presents loss of recent memory, spatial and temporal disorientation, and lack of interest in performing activities but can perform daily activities.

The moderate phase is characterized by marked and significant memory loss, and individuals require help to perform activities of daily living (ADLs) and IADLs. At this stage, the individual exhibits language disorders, irritability, and hallucinations [1].

In the severe phase, cognitive impairment is more significant, and memory loss is very marked, which prevents the performance of IADLs and ADLs [4]. In this phase, language changes are evident; the individual starts to communicate only by echolalia, has great difficulty recognizing faces and the environment where he/she lives, has difficulty controlling the sphincter and swallowing, and requires full assistance from caregivers [1].

Although pharmacological treatment is essential, in recent decades, there have been great advances in the treatment of AD, and nonpharmacological methods have been adopted to delay cognitive deficits and reduce functional disabilities [5]. Such methods include cognitive interventions (CI), physical activities, environmental restructuring, nutritional guidance, health guidance, psychological support for caregivers and family members, and therapeutic activities, such as group activities overseen by a multidisciplinary team [6].

Cognitive training (COG) associated with transcranial direct current stimulation (tDCS) has gained importance as an adjuvant treatment in AD [5]. Such training refers to practicing a set of standardized tasks to address cognitive functions such as memory, attention, and problem solving. Pencil-and-paper tasks, computer tasks, and daily activities can be used to train a specific skill, and this training can take place individually or in a group setting. In addition to reducing cognitive decline, COG can improve specific cognitive functions [7].

TDCS is a low-cost, portable, and safe tool capable of modulating cortical activity and inducing neuroplasticity mechanisms [8]. Neural activity is modulated using electricity without acting directly on neurons, which differs completely from electroconvulsive therapy [5]. TDCS is characterized by a low-intensity continuous electric current ranging from 0.5 to 2 mA.

This technique has been used to induce neural plasticity involving neural modulation through electrical current to minimize cognitive impairment and reduce the functional losses caused by AD [9]. In addition to the use of the active current, the device can generate a simulated or sham current that remains active for only 20–30 seconds and decreases gradually so that the sensations experienced by the individual are similar to those that occur with active current [10].

In this type of treatment, COG activities associated with tDCS are used to retain the ability to concentrate, sequence thoughts, maintain attention, and make active choices, and the individual is encouraged to use and preserve his/her skills; thus, it is a preventive and maintenance treatment [11].

People with AD have impairment in multiple cognitive domains, including episodic memory and executive functions. Kertesz and Mohs [12] define episodic memory as memories that store events experienced by the individual (biographical details). In general, difficulty with free recall and recognition is the first indication of a deficit. These deficits result in the loss of identity and independence, in addition to severely affecting social interactions with family and friends.

Executive functions refer to high-level cognitive processes related to setting goals and making appropriate choices in new situations, in addition to regulating actions and behaviors [13]. These functions are related to the frontal lobe, and individuals with disorders related to this area have difficulties planning and performing certain tasks and serious problems with controlling and regulating behavior [14].

The objective of this study was to verify the efficacy of COG associated with tDCS in episodic memory and executive functions in two subjects in the mild AD stage.

## Case presentation

### Case 1

Subject number 1 (S1), Brazilian Latin American woman, 82 years old, with high school education, was referred to the research center through the Associação Brasileira de Alzheimer (Brazilian Association of Alzheimer's Disease; ABRAZ). She was diagnosed with AD 8 years ago and used an Exelon patch for AD symptoms.

### Case 2

Subject number 2 (S2), Brazilian Latin American woman, aged 88 years, with an incomplete elementary education, was referred to the research center through the Centro de Atenção Integral à Saúde (Center for Comprehensive Health Care; CAIS). The patient was diagnosed with AD 1 year ago and received medical orientation to temporarily discontinue medications for AD.

Cognitive stimulation was applied concomitantly with neurostimulation, that is, the cognitive activities corresponded to the stimulated brain area. Anodic tDCS was applied to six different regions of the cortex: the dorsolateral prefrontal cortex bilaterally (F3 and F4), the somatosensory association cortex bilaterally (P3 and P4), and Broca’s (F5) and Wernicke’s (CP5) areas; placement was determined by the international 10 × 20 system of electroencephalogram (EGG) electrode placement based on the most frequent pattern used in studies with tDCS [10]: a current of 2 mA was applied for 10 minutes to each area three times a week for a total of 24 sessions. The cathode was positioned in the supraorbital area.

While the prefrontal cortex was being stimulated during tDCS, action- and object-naming activities and spatial memory tasks (shapes, colors, and letters) were performed as part of the COG—spatial attention activities (shapes and letters) were performed for the somatosensory association cortex, syntax and grammar activities were performed for Broca’s area, and activities for understanding lexical meaning and categorization were performed for Wernicke’s area (Table 1).

**Table 1:** Weekly stimulation protocol

First week	Protocol	Stimulated area
First session	A	Broca’s area (F5), dorsolateral prefrontal cortex (F4), and somatosensory association cortex (P3)
Second session	B	Wernicke’s area (PC5), dorsolateral prefrontal cortex bilaterally (F3), and somatosensory association cortex bilaterally (P4)
Third session	A	Broca’s area (F5), dorsolateral prefrontal cortex (F4), and somatosensory association cortex (P3)

A descriptive analysis was performed as described in Table 2, which shows the scores on the Alzheimer’s Disease Assessment Scale cognitive subscale (ADAS-Cog), the Word Recall task, and the Stroop test, Victoria version, before and after treatment.

**Table 2:** Results for both participants before and after treatment

Instrument	Subject	Before treatment	After treatment
ADAS-Cog			
Word recall	S1	5	4
	S2	5	4

Stroop test, Victoria version		C	P	I	C	P	I
Time (seconds)	S1	22	29	44	23	27	31
	S2	51	48	115	19	33	44
Errors	S1	0	0	2	0	0	1
	S2	1	0	2	0	0	2

*C* color, *P* words, *I* interference. Scores in seconds (s)

The comparison of the results of tests performed before and after the treatment period showed a 1-point improvement on the Word Recall task, a subtest of the ADAS-Cog instrument that evaluates the symptoms related to the decline of episodic memory, for both subjects.

Improvement in the executive function domain was also reflected by the subjects’ scores on the Stroop test, Victoria version. On the pretest, S1 took 22 seconds to complete card “C,” 29 seconds for card “P,” and 44 seconds for card “I,” and made two errors on the “I” card. On the posttest, S1 took 23 seconds for card “C,” 27 seconds for card “P,” and 31 seconds for card “I”; however, her time increased by 1 second on card “C,” and she made one error on card “I.”

On the Stroop test, Victoria version, pretest, S2 took 51 seconds for card “C,” 48 seconds for card “P,” and 115 seconds for card “I”; she made one error on card “C” and two errors on card “I.” After treatment, S2 took 19 seconds for card “C,” 33 seconds for card “P,” and 44 seconds for card “I,” and made two errors on card “I.”

These results indicate that both subjects improved equally in the episodic memory domain. In addition, both achieved improvements in executive functions because, for these instruments, lower scores indicate better performance. However, S2 had a more significant improvement in executive function than S1 did. In any case, the results presented here show that the symptoms of decline were reduced and the performance of the participants was improved with the application of COG concomitant with tDCS.

## Discussion and conclusions

The scores obtained on the Word Recall task of the ADAs-Cog subscale and the Stroop test, Victoria version, showed that the intervention had a positive impact on the executive functions and episodic memory domains, since changes occurred after the treatment period.

The participating subjects, S1 and S2, who underwent an intermittent protocol of anodic tDCS combined with COG for 2 months, showed positive therapeutic effects similar to those of subjects treated with pharmacological therapy for many months [8]. This fact corroborates a systematic review of studies on the use of cholinesterase inhibitors for AD, which found that individuals treated with this medication achieved an average improvement of 2.7 points in global cognitive function, as evaluated with the ADAS-Cog subscale, after 6–12 months of treatment [15].

A case report by Andrade *et al*. [8], in which adjuvant treatment with tDCS was administered to the dorsolateral prefrontal cortex with a current of 2 mA and the cathode located in the supraorbital region, found that after ten consecutive 30-minute sessions, a patient with AD obtained significant improvements in episodic memory. This improvement was verified by a neuropsychological evaluation in which the Word Recall task was administered before and after treatment.

S1 and S2 achieved positive results in the episodic memory and executive functions domains through the application of neurostimulation to the dorsolateral prefrontal cortex region. Similar results were found in a study conducted by Boggio *et al*. [16], who compared the efficacy of tDCS with anodic current to the left dorsolateral prefrontal cortex and the temporal cortex and obtained good results regarding declarative memory after the treatment period.

Pesente *et al*. [14] reviewed the literature on the effects of neuromodulation with tDCS on executive functions in healthy individuals; they found that the anodic current, the same current used in our study, was the most widely used. In one of their experiments, Pesente *et al*. [14] found that individuals who received anodic tDCS of the right lateral dorsal prefrontal cortex obtained greater gains in the performance of complex verbal tasks than those treated with the sham current. In the same review, Pesente *et al*. [14] wrote about a study on the effect of anodic and cathodic TDCS on planning abilities at 6 months and 1 year and found increased performance as a result of both types of stimulation.

According to Lopes, Bastos, and Argimon [17], COG is related to an increase in abilities in these functional areas, which prevents cognitive decline and consequently promotes better quality of life. Studies investigating the use of tDCS combined with COG have verified the efficacy of the two therapies combined. With the aim of evaluating the effects of noninvasive brain stimulation on cognitive function in healthy elderly individuals and elderly individuals with AD, a systematic review and meta-analysis of 11 studies with 200 patients by Hsu *et al*. [18] showed that neurostimulation during the execution of tasks had better effects than when neurostimulation and tasks were performed separately. The results of that study do not propose replacing traditional treatment; rather, they support the combined use of COG stimulation and tDCS as complementary and adjuvant treatments in AD to avoid the rapid decline caused by the disease and to favor the preservation of remaining functional areas for a longer time [8].

The two presented cases show that tDCS associated with COG is an effective adjuvant method for the treatment of patients diagnosed with mild AD, who can use its effects in their daily routines to reduce their cognitive deficits, keep intact areas active, and/or compensate for lost functions.

Research with large samples and a randomized and controlled design will be conducted to better understand the benefits of applying this treatment protocol. This will allow us to relate the COG variable to the use of tDCS, since our results were obtained through a sample of two cases and did not consider neuroimaging methods or biomarkers to evaluate cortical and subcortical activity.

## Data Availability

The datasets during and/or analyzed during the current study are available from the corresponding author on reasonable request.

## Abbreviations

AD: Alzheimer’s disease
IADL: Instrumental activity of daily living
ADL: Activity of daily living
CI: Cognitive intervention
COG: Cognitive training
tDCS: Transcranial direct current stimulation
mA: Milliampere
S1: Subject 1
ABRAZ: Brazilian Association of Alzheimer’s Disease
S2: Subject 2
CAIS: Center for Comprehensive Health Care
EGG: Electroencephalogram
